# The Neuroprotective Role of A2A Adenosine Purinoceptor Modulation as a Strategy Against Glioblastoma

**DOI:** 10.3390/brainsci14121286

**Published:** 2024-12-21

**Authors:** Júlia Leão Batista Simões, Geórgia de Carvalho Braga, Michelli Fontana, Charles Elias Assmann, Margarete Dulce Bagatini

**Affiliations:** 1 Medical School, Federal University of Fronteira Sul, Chapecó 89815-899, SC, Brazil; julialeaobatistasimoes@gmail.com (J.L.B.S.); braga.georgia18@gmail.com (G.d.C.B.); fontana.michelli@gmail.com (M.F.); 2 Department of Biochemistry and Molecular Biology, Federal University of Santa Maria, Santa Maria 97105-900, RS, Brazil; 3 Graduate Program in Medical Sciences, Federal University of Fronteira Sul, Chapecó 89815-899, SC, Brazil

**Keywords:** glioblastoma, Istradefylline, purinergic system, A2A purinoceptor, adenosine, Parkinson’s disease, neuroprotection

## Abstract

Glioblastoma (GBM) is a highly lethal type of cancer, frequently presenting an unfavorable prognosis. The current treatment options for this neoplasia are still limited, highlighting the need for further research evaluating new drugs to treat GBM or to serve as an adjuvant to improve the efficiency of currently used therapies. In this sense, the inhibition of A2A receptors in the brain has presented a neuroprotective role for several diseases, such as neurodegenerative conditions, and it has been suggested as a possible pharmacological target in some types of cancer; thus, it also can be underscored as a potential target in GBM. Recently, Istradefylline (IST) was approved by the FDA for treating Parkinson’s disease, representing a safe drug that acts through the inhibition of the A2A receptor, and it has also been suggested as an antineoplastic drug. Therefore, this work aims to explore the effects of A2A receptor inhibition as a therapy for GBM and assess the feasibility of this blockage occurring through the effects of IST.

## 1. Introduction

Recently, the Food and Drug Administration (FDA) in the United States approved the use of Istradefylline (IST) as an adjuvant medication in treating patients with Parkinson’s disease (PD) [1]. The drug’s main activity is its antagonist effect on the A2A receptor (A2AR) in the brain, thus reducing motor deficits, which are the most common symptoms reported by PD patients [1].

In this scenario, the previously known promising characteristics of the drug, along with new studies being developed, have led to the emergence of different hypotheses regarding the impacts of IST on other conditions linked to neurological impairment. It has been previously studied for its effects on certain dysfunctions, such as hearing loss [2], memory deficits [3], and restless legs syndrome [4]. In 2023, the drug was evaluated against alcohol-induced respiratory suppression [5] and melanoma [6], as well as for its potential as an adjuvant in therapy against neuroblastoma [7].

Considering the initial positive results related to the impact of IST in neuroblastoma, the drug’s potential against tumors deserves attention [7]. Evidence has been found regarding the role of this drug as a caffeine analog and its activity as an A2A receptor (A2AR) antagonist, which can indicate its possible effect on other types of cancer [6,8].

Glioblastoma (GBM) represents one of the most dangerous types of cancer, with a low response to a wide range of therapies [9]. This malignant brain tumor has high invasiveness and capacity for proliferation, making it extremely life-threatening [9]. Furthermore, studies indicate that GBM may respond to therapies related to the activity of A2AR, suggesting that future drugs that antagonize this receptor could be beneficial [10,11].

Therefore, the lack of therapies with expressive effectiveness against GBM and its aggressiveness indicates the need for further research on possible drugs to provide new perspectives in GBM treatment. Hence, this work offers insight into the possible effects of IST in this context.

## 2. Association Between Glioblastoma and A2AR

Different pathways contribute to the progression of GBM; among them, the molecular mechanism of hypoxia appears to promote its growth [12,13]. The immune system also plays a direct role in GBM development [13] by creating an immunosuppressive microenvironment [12,14] characterized by the recruitment of immunosuppressive cells, such as natural killer T (NKT) and T-suppressor cells like myeloid-derived suppressor cells (MDSCs) [15,16]. The release of immunosuppressive cytokines and chemokines, such as transforming growth factor-β (TGF-β), interleukin 10 (IL-10), and prostaglandin E2 (PGE2), further contributes to this immunosuppressive environment. Additionally, the hypoxic conditions in the GBM microenvironment increase the concentration of adenosine, a small nucleoside involved in blocking antitumor immunity. Studies have shown that adenosine inhibits the function of natural killer cells (NKCs) and impairs the ability of cytotoxic T cells to target tumor cells [11,17,18,19].

In the extracellular area, adenosine is produced from ATP after hydrolysis by specific ectoenzymes, namely CD39 (ecto-nucleoside triphosphate diphosphohydrolase 1, E-NTPDase1) and CD73 (ecto-5′-nucleotidase, Ecto5′NTase), which are expressed in microglial cells [20]. A study conducted in vivo demonstrated that mice lacking CD73 had lower levels of extracellular adenosine, suggesting that ATP degradation is the primary source of extracellular adenosine [21]. Because angiogenesis is essential for tumor cell growth, it was demonstrated that this process is blocked in mice deprived of CD39, leading to reduced tumor growth [22]. Thus, it is hypothesized that tumor-derived adenosine serves as a mechanism by which tumors escape the immune response [23], as the immune system fails to activate in the presence of antigens due to the inhibition of T cells by adenosine [24].

Studies have reported that extracellular adenosine is an essential regulator of several aspects of tumorigenesis, angiogenesis, tumor cell growth, and metastasis [25]. Kazemi and colleagues provide an interesting review of the expression of adenosine receptors in different tumor cell lines and their effects, including proliferative and tumor-protective expressions, after their activation [26]. Adenosine is expressed in various tissues and modulates the vasoconstriction and vasodilation of arteries and veins. Furthermore, it inhibits lipolysis, induces bronchoconstriction, and regulates diuresis, muscle tone, and locomotion [27]. At the CNS level, it exerts neuroprotective activity against ischemic events, hypoxia, and oxidative stress and modulates the release of neurotransmitters [28]. It is also involved in the regulation of cytokines and the production of T lymphocytes by the immune system [29].

**Figure 1:**
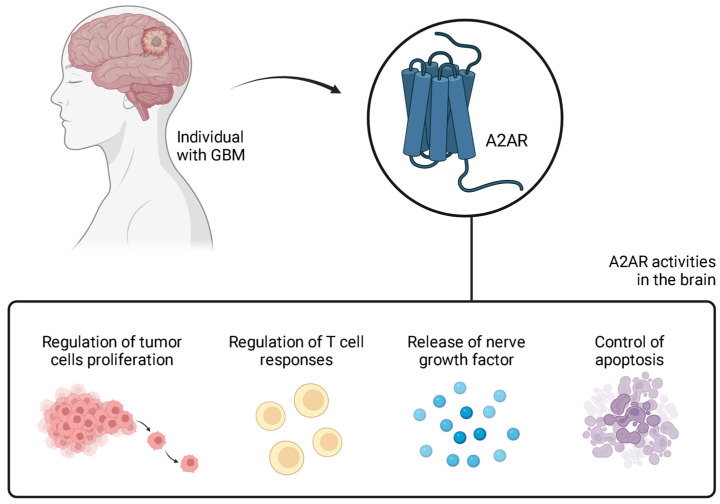
The role of A2AR in patients with GBM. A2AR can mediate various cellular and immune responses in the brains of individuals with GBM. This receptor regulates tumor cell proliferation, T cell responses, the release of nerve growth factor, and apoptosis. Hence, it can influence the development and growth of GBM. Figure made in BioRender (https://app.biorender.com/, accessed on 26 October 2024).

Adenosine is a purine nucleotide that acts in several physiological functions by interacting with its receptor subtypes (A1, A2A, A2B, and A3). A2A and A2B receptors are primarily expressed in the CNS, especially in presynaptic areas of the hippocampus, where they modulate the release of neurotransmitters, such as glutamate, acetylcholine, gamma-aminobutyric acid (GABA), and norepinephrine [30], as well as postsynaptic regions of the basal ganglia, where they influence neuronal plasticity. These receptors are also expressed on astrocytes and oligodendrocytes [30] and immune system cells [31], such as regulatory T cells, macrophages, and natural killer cells (NKCs) [32], suggesting that they could be valid candidates for cancer immunotherapy. Both in vitro and in vivo studies have shown the presence of adenosine receptors on microglia, including the expression of A2AR [33] (Figure 1).

From this perspective, stimulation of A2AR in rat microglia induces the expression of nerve growth factor and its release, thus exerting a neuroprotective effect [32,33]. At the same time, it induces the expression of cyclooxygenase-2 (COX-2) in rat microglia, releasing prostaglandins [34]. Other studies using A2AR antagonists have inhibited tumor growth, reduced CD4+ and regulatory T cells, and improved antitumor T cell responses [35]. There is conflicting evidence regarding adenosine-receptor-mediated actions on GBM proliferation. In GBM stem cells, activation of A1AR and A2BR appears to reduce tumor proliferation and induce apoptosis [36], whereas in non-GBM stem cell lines, activation of A1, A2B, or A3 receptors induced increased proliferation. Liu and collaborators [36] reported a pro-proliferative action of adenosine mediated by A2B receptor activation in GBM cell lines subjected to hypoxia.

In the study by Kim and Bynoe [27], mice lacking CD73 and unable to synthesize extracellular adenosine have a more cohesive blood–brain barrier (BBB) and are protected against experimental autoimmune encephalomyelitis (EAE), the animal model for multiple sclerosis. More recently, it was shown that activation of A2AR with a broad-spectrum agonist or an FDA-approved specific agonist, N-Methyl-2-[4-[(methylamino)carbonyl]-1H-pyrazol-1-yl]adenosine (Regadenoson), increased drug accumulation in the brain in a time- and dose-dependent manner [37,38]. BBB opening under AR signaling was reversible. Thus, the duration of BBB permeability depended on the half-life of the AR agonist. Similarly, signaling activation was shown to exert its effects in the paracellular pathway, altering VE-cadherin and claudin-5 expression to promote BBB permeability in primary human brain endothelial cells [37,39]. Therefore, evaluating the therapeutic potential of adenosine receptor modulators may be the future of combating GBM.

## 3. Istradefylline: An Overview

Istradefylline (IST) is a selective adenosine receptor modulator approved in 2019 by the U.S. FDA and indicated as an adjunctive treatment to levodopa/carbidopa for adults with PD who experience “off” episodes [40]. This medication is primarily metabolized by cytochrome P450 (CYP) 1A1 and 3A4, and exposure to IST is affected by smoking and the use of CYP3A4 inhibitors [41]. The most common adverse reactions (occurring in at least 5% of patients and more frequently than with placebo) with IST use are dyskinesia, dizziness, constipation, nausea, hallucinations, and insomnia [40]. The recommended dosage for this medication is 20 mg orally once a day, which can be increased up to 40 mg daily [40]. In this section, we will discuss the action of IST on the A2AR, its indication in treating PD, and its potential for treating other health conditions (Table 1).

IST acts as an A2AR antagonist [41,46]. The A2AR is a protein that belongs to a family of four G protein-coupled receptor (GPCR) adenosine receptors, which regulate several pathophysiological conditions in the central and peripheral nervous system [43]. These A2ARs are primarily located in the basal ganglia on the external surfaces of neurons in the indirect tracts between the striatum, the external globus pallidus, and the substantia nigra [41,47]. An antagonistic relationship between adenosine and dopamine is present because A2ARs are co-localized with dopamine D2 receptors in the striatum [47].

Thus, activation of the A2AR reduces the affinity of dopamine D2 receptors for dopamine agonists, leading to a decrease in mobility, which is beneficial for treating patients with PD [41,47]. Therefore, therapies involving the activation of dopamine D2 receptors and inhibition of A2AR through therapies, such as IST, contribute to improving symptoms of movement dysfunction in PD patients [41,47]. Additionally, adenosine antagonists have a neuroprotective effect on dopaminergic neurons affected by PD. Moreover, activation of the A2AR affects GABAergic transmission by inhibiting it in the striatum and increasing it in the globus pallidus, which can be corrected with the use of IST [41,47].

Given these therapeutic effects, IST is used in combination with levodopa/carbidopa in adult patients with PD who experience “off” episodes [41]. These episodes are characterized by an increase in symptoms and occur unexpectedly, impacting the patient’s quality of life, partly due to levodopa therapy [41]. IST promotes dopaminergic activity by antagonizing adenosine in the basal ganglia [41]. Thus, by targeting A2AR in the basal ganglia, IST decreases the duration of “off” episodes in patients with PD, providing additional neuroprotective effects [46]. Furthermore, it neutralizes the side effects of levodopa by reversing motor disability during “on” periods without causing dyskinesia [46]. In relation to therapies for PD, Torres-Yaghi and colleagues [48] conducted a post hoc analysis of the effect of treatment with IST as an adjunct to levodopa treatment. Their study concluded that it could improve the motor skills of PD patients with motor fluctuations, both in patients with the tremor-dominant subtype of the disease and in the subtype of postural instability and gait difficulty [48]. Considering the positive effects observed in the adjunctive treatment of levodopa therapy in PD patients, other therapeutic possibilities are being considered for the medication. IST is also being evaluated as a potential monotherapy in PD patients with cognitive impairment in phase two studies and in patients with amyotrophic lateral sclerosis, spinal cord injuries, and myelopathy in phase one studies [46]. Furthermore, studies are targeting the protection of the kidneys of patients undergoing cisplatin treatment [42].

Cisplatin is a chemotherapy drug used in the treatment of various cancers, but it often causes severe adverse effects, such as nephrotoxicity and peripheral neuropathy [42]. The adenosine A2AR also controls renal pathologies, such as ischemia–reperfusion injury, fibrosis, diabetic nephropathy, and glomerulonephritis [42]. Dewaeles and collaborators [42] conducted a study using rat models with acute, subchronic, and chronic cumulative administration of cisplatin. They found that IST mitigated cisplatin-induced nephrotoxicity and pain hypersensitivity, while also enhancing the antitumor properties of cisplatin [42].

Another critical aspect of the therapeutic potential of adenosine A2AR antagonists is their potential to serve as a new strategy to combat neurodegenerative diseases, such as Alzheimer’s [43]. A2ARs in the brain are mainly located in glutamatergic synapses, where their activation at the presynaptic level leads to an increase in glutamate release, contributing to excitotoxicity [43]. Thus, the involvement of the adenosine A2AR in glutamatergic synaptic physiology is related to Alzheimer’s, and A2AR antagonism may help reduce hippocampus-dependent memory impairment, providing a way to combat synaptic toxicity. Furthermore, inhibiting A2AR-mediated cytokine production could decrease neuroinflammation and potentially reduce memory loss [43].

Blocking the A2AR has been shown to improve human memory, suggesting a possible strategy to address cognitive deficits in Alzheimer’s patients [43]. In this sense, low doses of IST have been shown to improve spatial memory and habituation in animal models of Alzheimer’s, indicating that drugs targeting the A2AR, like IST, hold promise for treating dementia [43]. A2B antagonists and mixed A2A/A2B antagonists may also have neuroprotective properties and are also being explored for their potential applications in cancer immunotherapy [43,44,45]. One of the main challenges in modulating the adenosine receptor for cancer treatment is interrupting tumor growth, as it does not directly impact gene expression suppression or induction [44]. However, an important aspect to consider is that adenosine, through the A2AR, responds to tissue injury by promoting angiogenesis [45]. Thus, considering that angiogenesis is a crucial factor for tumor metastasis, A2A antagonists could play a role in negatively regulating tumor growth by blocking angiogenesis and metastasis [45]. Therefore, the most likely scenario is that the activation or blockade of adenosine receptors could serve as an adjuvant therapy in chemotherapeutic and immunotherapeutic treatments [44].

In this sense, adenosine, its affinity receptors, and related enzymes are relevant in the action of the immune system, as there is a high concentration of adenosine in the tumor microenvironment, which hinders the activation of lymphocytes capable of destroying tumor cells [44]. In this regard, a selective A2AR antagonist reduces cAMP levels, enabling lymphocytes to fight tumor cells [44]. Therefore, A2AR antagonists may be a strategy to overcome the immunosuppressive effects of adenosine in the tumor microenvironment [45]. Consequently, several clinical trials are underway to test the combination of A2AR antagonists with anticancer immunotherapies and the addition of inhibitors of the enzyme that converts AMP to adenosine (CD73) to these trials [44].

## 4. The Potential Role of Istradefylline Against Glioblastoma

GBM multiforme is the most prevalent and aggressive form of tumor affecting the adult brain. Due to its aggressive nature and infiltrative growth pattern, it remains one of the most challenging tumors to manage [49]. The standard treatment for GBM currently includes surgical intervention to achieve total resection, followed by a combination of chemotherapy and radiotherapy [49].

Despite advancements in surgical techniques and intraoperative technology, the prognosis remains poor. In this scenario, with current medical treatment, patients have a median overall survival of 15 months, with only 5% of patients living longer than 5 years [50]. Considering the limited survival rates and the challenges of current therapies, exploring alternative approaches for the disease is crucial to improve patient outcomes. Efforts to integrate immunotherapies with conventional chemotherapy and radiotherapy in GBM have proven ineffective [49]. This limited success is attributed to the tumor’s ability to evade immune detection by developing various immunoresistance mechanisms [49].

Adenosine receptors, notably the A2A subtype, have gained interest as potential targets for treating various neurodegenerative disorders linked to neuroinflammation, such as Parkinson’s and Alzheimer’s diseases, as well as multiple sclerosis [50]. In this regard, the approval of Istradefylline, an A2AR antagonist, as an adjunct therapy for Parkinson’s disease opens up new therapeutic possibilities [50]. Inhibiting A2AR has been shown to provide neuroprotective benefits by mitigating neuroinflammatory processes and reducing astroglial and microglial activation [50]. These findings suggest that targeting A2AR is a promising therapeutic approach for GBM.

Recently, a few studies have suggested the potential role of IST as an antineoplastic drug, either highlighting the need for a deeper study of this molecule’s behavior in this context or presenting positive results of its use in some tumors [6,7,11,51]. In this regard, little is known about its antitumor effects in the central nervous system (CNS); however, in other tissues, research suggests that blocking A2AR has essential effects on inflammatory responses. This includes decreasing malignant cell proliferation and metastasis formation, increasing early apoptosis, and inducing a better response to chemotherapy [6].

Studies indicate that the P1 family of receptors is responsible for inducing an immunosuppressive response in the tumor microenvironment, inhibiting the activities of immune cells, such as natural killers and cytotoxic T cells, for example [11,52]. The activity of A2AR, in particular, has been linked to immune evasion of malignant cells and the development of metastasis, which is closely associated with cancer progression [11,53]. Furthermore, in vitro studies with GBM cells have suggested that excessive adenosine interaction with A2AR protects the tumor, including protection against chemotherapy, and thus reducing tissue response to it [11,54]. Hence, blocking A2AR with IST has been indicated as a potential strategy against GBM by inducing immune responses against the tumor, enhancing the response to standard chemotherapy, and providing new perspectives for treating patients who receive this unfavorable diagnosis.

When considering the association of this drug with A2AR, other characteristics of IST also support its potential as a pharmacological target against GBM. In this sense, IST represents a modified methylxanthine, a group of purine-derived xanthine derivative substances that can be found in nature or produced synthetically known for their A2AR antagonistic effect [8]. Recently, this group of molecules has been studied for its protective effects against neurodegenerative conditions, showing promising results, such as IST itself in PD, for example [8].

Additionally, several different methylxanthines have been studied for their antitumoral effects as well, including tumors of the CNS. In a study with neuroblastoma cell lines, Tran and colleagues [7] evaluated the potential of caffeine as an adjuvant therapy against the tumor. As a result, caffeine could improve the anti-growth effect of statins over malignant cells, demonstrating a positive effect against cancer [7]. Initially, caffeine reduced the activity of the mevalonate pathway, which generates sterols, precursors of various molecules, including cholesterol, a structural component of cellular membranes [7]. Sterols can also form several other molecules necessary for maintaining intracellular functions. Hence, reducing their availability using statins and reducing the activity of the mevalonate pathway using caffeine represent relevant measures to prevent tumoral progression [7].

Moreover, Tran and colleagues [7] evaluated the property of caffeine that allows for the reduction of mevalonate pathway activities and found that its ability to inhibit A2AR was responsible for this phenomenon [7]. Additionally, they tested other A2AR inhibitors, finding the same type of activity, including IST. Therefore, the drug can affect cellular lipid metabolism through A2AR inhibition, ultimately preventing cell proliferation.

Furthermore, other studies have also found that caffeine induces apoptosis in malignant cells [55]. According to these studies, apoptosis has been mediated by this substance in several types of cancer, including GBM [56,57,58]. Different pathways have been suggested as responsible for the influence of this substance on the cellular cycle and apoptosis, with the modification of the transcription of some genes, mainly FOXO1, being considered a possibility [59]. In this regard, a study with mice evaluating the autophagy and apoptosis of chondrocytes has suggested that administering A2AR agonists is also responsible for modifying FOXO1 expression, but it leads to the reduction of apoptosis [60]. Although this association is not completely clear, A2AR antagonism can induce apoptosis and avoid cancer progression [60].

Another methylxanthine that deserves attention in the IST scenario is theobromine. Sugimoto and colleagues conducted studies on the effects of this molecule on GBM cell lines, finding that it inhibits the proliferation of malignant cells [61]. According to their study, the molecule can affect PDE4, ERK, Akt/mTOR, NF-κB, p38-MAPK, and JNK pathways, reducing proliferative activities and inducing apoptosis [61]. Despite the absence of scientific literature linking this behavior to A2AR modulation, studies suggest that caffeine and IST can also affect some proliferative and apoptotic pathways through A2AR inhibition under pathological conditions [6,62]. Thus, IST can potentially influence these pathways, and it is a promising drug against GBM.

**Figure 2:**
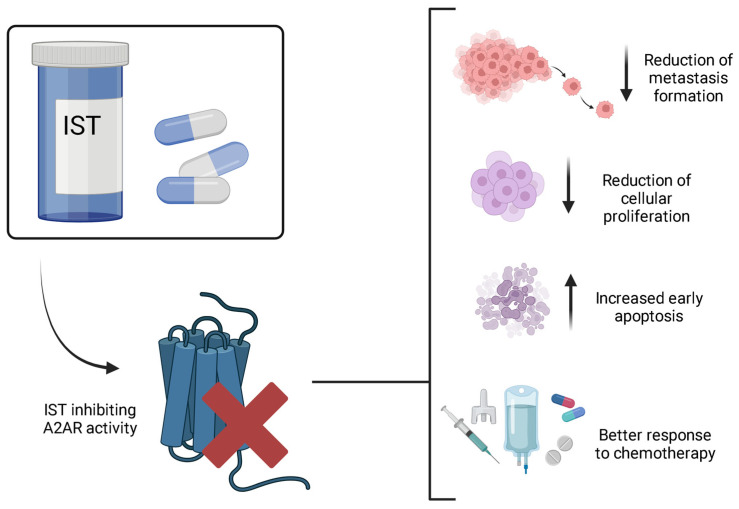
The effects of IST on tumor development. IST acts by inhibiting the activities of A2AR, leading to a reduction in metastasis formation and cellular proliferation. Additionally, antagonism to this receptor also increases early apoptosis and improves responses to chemotherapy, contributing to the treatment of the disease. Figure made in BioRender. (https://app.biorender.com/, accessed on 26 October 2024.).

Finally, in the study by Da Silva and colleagues, IST was shown to exhibit pro-inflammatory behavior in the tumor microenvironment of mice with melanoma [6]. A2A antagonism increased the expression of P2X7 receptors (P2X7R) and blocked tumor growth pathways [6]. Activation of P2X7R is responsible for mediating inflammatory responses and immunological activities by releasing cytokines, recruiting immune cells, and activating the NLRP3 inflammasome [60]. In the context of melanoma, this response is promising, as it allows individuals to activate their immune system to induce inflammation and fight cancer, helping to control the disease and prevent its progression. This feature could also be beneficial in the treatment of GBM (Figure 2).

In addition, there is an increased risk of thrombotic events in high-grade gliomas. Thus, one of the difficulties of treatment is associated with managing anticoagulant therapy, given the high background rate of intralesional bleeding. Studies indicate that despite the increased thrombotic risk, patients with GBM benefit from more extensive antithrombotic prophylaxis based on evaluating the use of heparin without considering the new oral anticoagulants [63]. It is worth noting that there are currently no protocols indicating or contraindicating prolonged antithrombotic prophylaxis considering that administering anticoagulants to these patients involves an increased risk of intracranial bleeding [63].

Additionally, studies suggest that the presence of inflammatory environments can affect the activity of cellular proliferative and apoptotic pathways, leading to increased apoptosis and reduced cell replication [64,65]. Therefore, the pro-inflammatory conditions induced by the use of IST through its A2AR antagonistic activity can also be responsible for regulating pathways that stimulate or halt the cell cycle. In this regard, such activity can be valuable in controlling tumor growth and infiltration, representing a promising mechanism of the drug.

Therefore, IST has extensive potential to serve as an antineoplastic drug or adjuvant in the GBM scenario. This molecule has shown promising results when analyzed as a therapy against other types of tumors. However, it also shares characteristics with other substances already evaluated in the GBM scenario, specifically the methylxanthines group to which IST belongs as a synthetic form of these molecules [8].

Furthermore, IST has already been proven as a safe drug capable of crossing the BBB and reaching the brain, indicating that it would be suitable for the treatment of GBM, a cancer with such an unfavorable prognosis. Moreover, this drug has shown several relevant activities against GBM. However, its proinflammatory effects in the tumor microenvironment are the primary pathway for developing its antineoplastic activities, indicating the need to create a hostile condition to combat GBM and the critical potential of IST in this context.

## 5. Conclusions and Future Perspectives

Evidence indicates that A2AR activity is associated with cancer progression, particularly metastasis. Blocking this cancer feature could lead to improved outcomes and potentially open up new therapeutic options. In this sense, taking into account the potential positive effect of blocking A2AR in inflammatory responses and the neuroprotective effects of A2AR inhibitors in neurodegenerative conditions, it is believed that IST has the potential to improve the response to chemotherapy in GBM patients.

The hypothesis that IST could inhibit cell proliferation by modulating A2AR is supported by the positive effect against cancer observed from A2AR inhibition in the testing of caffeine, another methylxanthine in neuroblastoma cells. Furthermore, A2AR antagonism may be able to induce apoptosis, which contributes to controlling cancer progression. This association with the pro-inflammatory behavior resulting from A2A antagonism boosts P2X7 receptor expression, enabling the immune system to induce inflammatory responses and fight cancer, thus controlling tumor growth. Given this positive evidence, it is suggested that the antitumor effects of IST on the central nervous system (CNS) be further investigated to offer new treatment approaches for GBM patients.

## Figures and Tables

**Table 1:** Therapeutic potential of IST for new treatments for health conditions.

Potential Use of IST	Therapeutic Effect	References
Adjuvant treatment with cisplatin	Mitigation of cisplatin-induced nephrotoxicity and pain hypersensitivity; enhancement of the antitumor properties of cisplatin	[42]
Treatment for Alzheimer’s and dementia	Reduction of hippocampus-dependent memory impairment; combating synaptic toxicity; reducing neuroinflammation	[43]
Adjuvant treatment to chemotherapy	Blocking angiogenesis; reducing tumor proliferation	[44,45]
Adjuvant treatment to immunotherapy	Reduction in cAMP levels enables lymphocytes to fight tumor cells	[44,45]

## Data Availability

No new data were created or analyzed in this study.
